# Mechanisms of Action of MiRNAs and LncRNAs in Extracellular Vesicle in Atherosclerosis

**DOI:** 10.3389/fcvm.2021.733985

**Published:** 2021-10-08

**Authors:** Hui Xu, Yu-Qing Ni, You-Shuo Liu

**Affiliations:** ^1^Department of Geriatrics, The Second Xiangya Hospital, Central South University, Changsha, China; ^2^Institute of Aging and Age-related Disease Research, Central South University, Changsha, China

**Keywords:** extracellular vesicles, microRNA, long non-coding RNA, endothelial cells, vascular smooth muscle cells, atherosclerosis

## Abstract

Atherosclerosis, a complex chronic inflammatory disease, involves multiple alterations of diverse cells, including endothelial cells (ECs), vascular smooth muscle cells (VSMCs), monocytes, macrophages, dendritic cells (DCs), platelets, and even mesenchymal stem cells (MSCs). Globally, it is a common cause of morbidity as well as mortality. It leads to myocardial infarctions, stroke and disabling peripheral artery disease. Extracellular vesicles (EVs) are a heterogeneous group of cell-derived membranous structures that secreted by multiple cell types and play a central role in cell-to-cell communication by delivering various bioactive cargos, especially microRNAs (miRNAs) and long non-coding RNAs (lncRNAs). Emerging evidence demonstrated that miRNAs and lncRNAs in EVs are tightly associated with the initiation and development of atherosclerosis. In this review, we will outline and compile the cumulative roles of miRNAs and lncRNAs encapsulated in EVs derived from diverse cells in the progression of atherosclerosis. We also discuss intercellular communications *via* EVs. In addition, we focused on clinical applications and evaluation of miRNAs and lncRNAs in EVs as potential diagnostic biomarkers and therapeutic targets for atherosclerosis.

## Introduction

Atherosclerosis refers to a complex chronic inflammatory disease that involves endothelial dysfunction, mononuclear cell accumulation, coagulation and thrombosis, cell proliferation and migration, as well as lipid deposition (1). Numerous cells, including endothelial cells (ECs), vascular smooth muscle cells (VSMCs), monocytes, macrophages, platelets and mesenchymal stem cells (MSCs) are involved in the initiation and progression of atherosclerosis (2). Globally, atherosclerosis is the major underlying cause of cardiovascular diseases, cerebrovascular diseases and peripheral vascular diseases, and is strongly associated with high morbidity and mortality rates (3). Therefore, studies should aim at identifying novel diagnostic tools and innovative therapies to delay or curb the development of atherosclerosis and prevent adverse complications, such as stroke and myocardial infarction.

**Figure 1:**
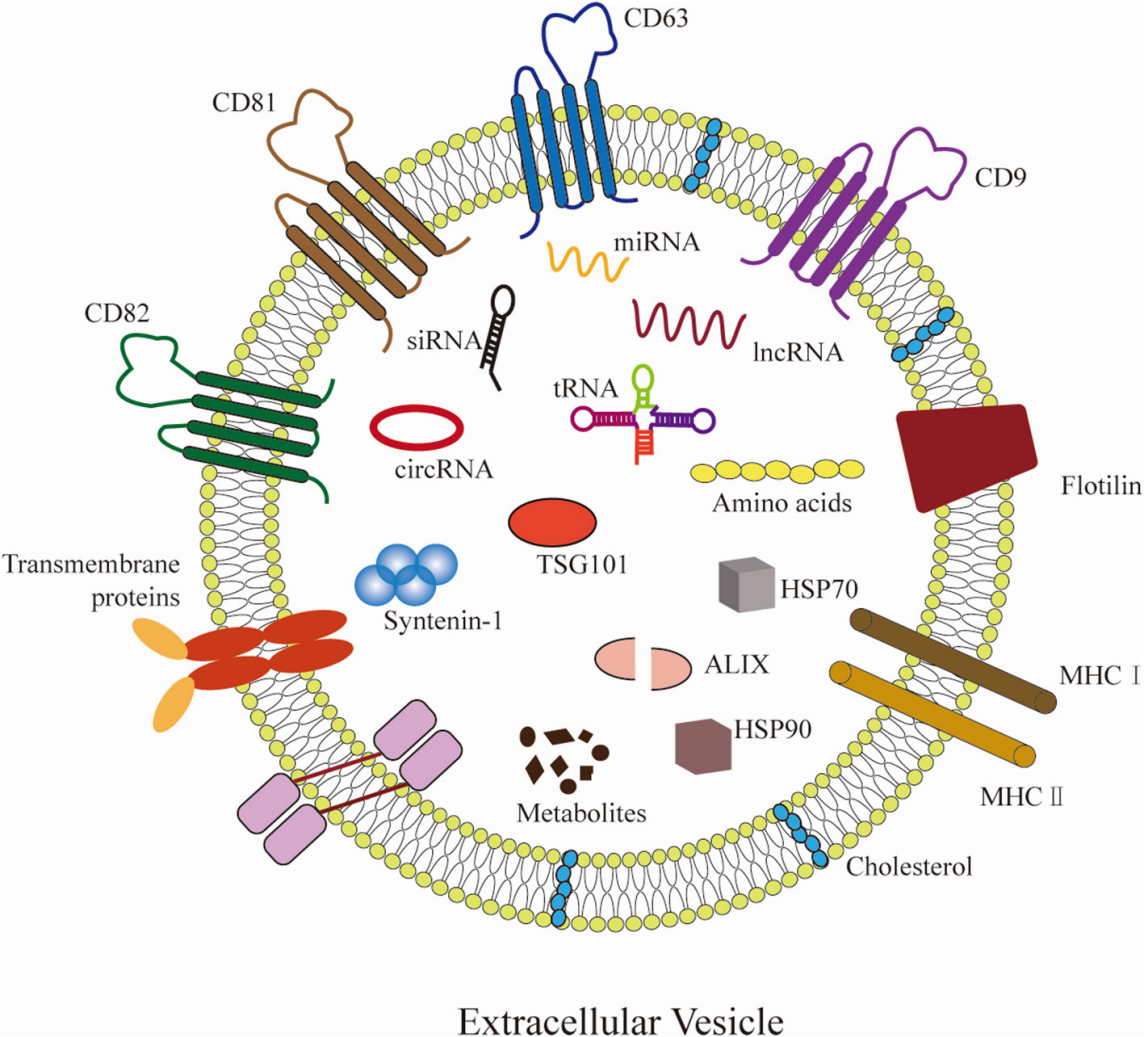
Hallmarks of EVs. EVs can be secreted by almost all cells. EVs carry various molecular cargos, including proteins, lipids, metabolites, and nucleic acids (mRNAs, miRNAs, lncRNAs, etc.). Proteins include tetraspanins (CD9, CD63, CD81, and CD82), ESCRT proteins (such as ALIX and TSG101), and syntenin-1 have emerged as commonly used markers for EVs. Some molecules are carried on the surface of EVs, including major histocompatibility complex (MHC) class I/II molecules, flotillin, and transmembrane proteins, etc.

Extracellular vesicles (EVs) originate from diverse cells are a heterogeneous group of cell-derived membranous structures, with an average diameter range from 20 to 1,000 nm (4–6). Prompted by the International Society of Extracellular Vesicles (ISEV), EVs are a general term of various subtypes of membrane structures released by cells. Accordingly, EVs include exosomes (50–150 nm in diameter) (7), ectosomes (ranging from 100 to 350 nm in diameter) (8), microvesicles (100 nm to 1 μm in diameter) (9), apoptotic bodies (ranging from 500 nm to >2 μm in diameter) (10), oncosomes (1 μm to up to 10 μm in diameter) (11), and other non-defined EV subtypes (12). EVs contain various bioactive molecules, including glycoconjugates, proteins, lipids, mRNAs, and non-coding RNAs, such as miRNAs and lncRNAs (13). In addition, exosome markers, including CD9, CD63, CD81, flotillin-1 and 2, ALIX, TSG101, and syntenin-1 are not strictly specific to exosomes (14–16) (Figure 1). Throughout this review the different types of vesicles will be referred to as EVs. It has been shown that EVs emerge as pivotal modulators of gene transcriptional expression and intercellular communication (5). Notably, emerging publications indicated that EVs released by multiple cell types are implicated in the pathogenesis and progression of atherosclerosis *via* miRNAs and lncRNAs delivery.

MiRNAs are endogenous single-stranded RNAs, composed of 20–25 nucleotides, which are post-transcriptional modulators that exhibit a critical role in modulating gene expression and protein synthesis (17). LncRNAs are defined as a class of long non-coding RNAs with a length of more than 200 nucleotides (18). Interestingly, it has been reported that both exosomal miRNAs and lncRNAs are implicated in atherosclerosis progression by affecting the function of different cells, including ECs, VSMCs, and immune cells. Although extensive research has been carried out on the EVs containing miRNAs and lncRNAs in atherosclerosis, the latent mechanisms are still not well-clarified.

In this review, we will focus on the cumulative roles of miRNAs and lncRNAs packaged in diverse cell types-derived EVs during atherosclerosis development. We also discuss cell-to-cell communications *via* EVs and miRNAs and lncRNAs delivery. In addition, we provided a comprehension of clinical applications and evaluation of miRNAs and lncRNAs in EVs as potential diagnostic biomarkers and therapeutic targets for atherosclerosis, focusing on recent developments in the field.

## MiRNAs and lncRNAs in EVs From Different Cells in Atherosclerosis

Endothelial dysfunction, VSMCs proliferation and migration, and immune cells infiltration are tightly associated with atherosclerosis progression. EVs can be released from almost all cell types, including ECs, VSMCs, monocytes, macrophages, DCs, platelets, and MSCs (19). A host of data supports that EVs play significant roles in the regulation of occurrence and development of atherosclerosis by delivering miRNAs and lncRNAs to recipient cells (20).

### MiRNAs in EVs From Different Cells in Atherosclerosis

MiRNAs are a sort of non-coding RNAs, which mediate protein synthesis through their interactions with messenger RNAs during atherosclerosis (21). Increasing evidence demonstrates that miRNAs packaged in EVs can modulate atherosclerosis through regulating inflammation, VSMCs proliferation and migration, mononuclear macrophage adhesion, as well as platelets activation. In this part, we summarize the emerging roles of miRNAs in EVs from different cells in atherosclerosis (Table 1).

**Table 1:** Function of miRNAs in EVs in atherosclerosis.

**Donor cells**	**MiRNAs**	**Expression**	**Recipient cells**	**Target /pathway**	**Effects**	**References**
ECs	miR-10a	↑	Monocytes	IRAK4 /NF-κB	Inhibit atherosclerotic lesion development through inhibiting inflammation	(22)
	miR-19b	↑	ECs	TGF-β2↓	Inhibit the development of atherosclerosis	(23)
	miR-126	↓	ECs	SPRED1	Promote atherosclerosis development	(24)
		↑	Macrophages	CXCL12↑	Inhibit atherosclerosis development by reducing inflammation	(25)
	miR-126-3p	↓	THP-1 cells	ICAM-1↑	Promote atherosclerosis development	(26)
	miR-143/145	↑	VSMCs	KLF2	Reduce atherosclerotic lesion formation	(27)
	miR-155	↑	ECs	eNOS↓	Promote atherosclerosis development by suppressing the endothelium vasorelaxation	(28)
	miR-210	↑	Macrophages	HIF-1	Reduce atherosclerotic lesion	(29)
	miR-222	↓	ECs	ICAM-1↑	Promote atherosclerosis development	(30)
	miR-505	↑	Neutrophils	NF-κB, SIRT3	Accelerate atherosclerosis progression in atherosclerosis mice by inducing NET formation	(31)
VSMCs	miR-92a miR-195	↑	Macrophages	IL-6↑, CXCL1↑	Stimulate atherosclerosis progression	(32)
	miR-133a	↓	VSMCs	IGF-1R↓	Promote atherosclerosis by suppressing VSMCs development	(33)
		↑	CPCs	*Bim, Bmf*	Improving cardiac function in a rat myocardial infarction model	(34)
	miR-150	↑	ECs	VEGF-A/VEGFR/PI3K/Akt	Inhibit atherosclerosis progression	(35)
	miR-155	↑	ECs	KLF5	Promote atherosclerosis progression through inhibiting the expression of endothelial tight junction protein	(36)
	miR-221/222	↑	HUVECs	PTEN/Akt	Inhibit atherosclerosis progression through repressing HUVECs autophagy	(37)
Monocyte/ macrophage	miR-21-3p	↑	VSMCs	PTEN	Accelerate atherosclerosis	(38)
	miR-99a miR-146b miR-378a	↑	Macrophages	NF-κB/TNF-α	Inhibit atherosclerosis progression by suppressing inflammation	(39)
	miR-106a-3p	↑	VSMCs	Caspase	Inhibit atherosclerosis progression through promoting cell viability and inhibiting cell apoptosis	(40)
	miR-146a	↑	Macrophages	IGF2BP1↓, HuR↓	Accelerate the development of atherosclerosis	(41, 42)
		↑	Neutrophils	SOD2↑		
	MiR-150	↑	HMEC-1	c-Myb↓	Promote atherosclerosis development by regulating ECs migration	(43)
	miR-155	↑	ECs	MyD88/NF-κB	Inhibit atherosclerosis progression	(44)
		↑		Sirt1/AMPKα2, RAC1/ PAK2	Aggravate myocardial injury by reducing the angiogenic ability of ECs	(45)
	miR-222	↑	VSMCs	CDKN1B, CDKN1C	Accelerate the development of atherosclerosis	(46)
	MiR-223	↑	Macrophages	Pknox1	Inhibit atherosclerosis by suppressing inflammation	(47)
DCs	miR-16, miR-21	↑	HUVECs	TNF-α↓, NF-κB	Inhibit atherosclerosis progression by suppressing TNF-induced endothelial inflammation	(48)
	miR-146a	↑	DCs, ECs	IRAK-1↓ TNF-α↓, IL-6↓	Inhibit atherosclerosis progression through modulating inflammatory responses	(49)
	miR-146a	↓	BMDCs	IRAK1, TRAF6	Accelerate the development of atherosclerosis by promoting inflammation	(50)
	miR-155	↑		TNF-α↑, IL-6↑		
	miR-203-3p	↓	BMDMs	cathepsin S↑	Accelerate development of atherosclerosis by downregulating Ctss in mice	(51)
Platelets	miR-25-3p	↑	ECs	NF-κB	Inhibit atherosclerosis progression by inhibiting endothelial inflammation	(52)
	miR-126	↑	ECs	VEGF↑, bFGF↑, TGF-β1↑	Accelerate atherosclerotic plaque growth, intraplaque hemorrhage, and thromboembolic events	(53)
	miR-142-3p	↑	ECs	BCLAF-1	Inhibit atherosclerosis development	(54)
	miR-223	↑	ECs	IGF-1R↓	Promote the development of atherosclerosis	(55)
		↑	HUVECs	ICAM-1↓	Inhibit atherosclerosis progression	(56)
	miR-223, miR-21, miR-339	↑	VSMCs	PDGFRβ↓	Promote atherosclerosis progression	(57)
	miR-320b	↑	ECs	ICAM-1↓	Inhibit atherosclerosis progression	(58)
MSCs	miR-30b	↑	HUVECs	DLL4↓	Inhibit atherosclerosis progression	(59)
	miR-31	↑	HUVECs	HIF-1↓	Inhibit atherosclerosis progression	(60)
	miR-125a	↑	ECs	DLL4↓	Inhibit atherosclerosis progression through promoting angiogenesis	(61)
	miR-125b	↑	VSMCs	Myo1e	Inhibit atherosclerosis progression	(62)
	miR-125b-1-3p	↑	Lymphocytes	BCL11B↓	Alleviate atherosclerosis	(63)
	miR-125b-5p	↑	N/A	Map4k4↓	Inhibit atherosclerotic plaque formation	(64)
	miR-126	↑	ECs, MSCs	PI3K/Akt/eNOS	Ameliorate atherosclerosis	(65)
	miR-145	↑	HUVECs	JAM-A↓	Reduce atherosclerotic plaque *in vivo*	(66)
	miR-223	↑	Macrophages	NLRP3	Inhibit rupture of atherosclerotic plaques	(67)
	miR-301	↑	Myocardial	N/A	Protect myocardial infarction by inhibiting myocardial autophagy	(68)
	miR-342-5p	↓	HUVECs	PPP1R12B↑	Alleviate atherosclerosis by promoting HUVECs apoptosis	(69)
	miR-512-3p	↑	ECs	Keap1	Inhibit atherosclerosis through repressing ox-LDL induced ECs dysfunction	(70)
	miR-let7	↑	Macrophages	HMGA2/ NF-κB	Attenuate the progression of atherosclerosis in ApoE(-/-) mice through regulating infiltration and polarization of M2 macrophage	(71)
				IGF2BP1/PTEN		

*ECs, endothelial cells; HUVECs, human umbilical vein endothelial cells; VSMCs, vascular smooth muscle cells; DCs, dendritic cells; BMDMs, bone marrow-derived macrophages; CPCs, cardiac progenitor cells; NF-κB, nuclear factor-kappa B; SPRED1, sprouty-related, EVH1 domain-containing protein 1; ICAM-1, intercellular adhesion molecule-1; STAT5, signal transduction and activator of transcription 5; IL-3, interleukin-6; KLF2, Krüppel-like factor 2; eNOS, endothelial Nitric oxide synthase; HIF-1, hypoxia-inducible factor−1; ATM, ataxia telangiectasia mutated; CXCL1, CXC chemokine ligand 1; IGF-1R, insulin-like growth factor-1 receptor; VEGF-A, vascular endothelial growth factor-A; PTEN, phosphatase and tensin homolog deleted on chromosome ten; TNF-α, tumor necrosis factor-α; bFGF, basic Fibroblast Growth Factor; BCLAF-1, Bcl-2-associated transcription factor-1; IGF2BP1, insulin-like growth factor 2 mRNA-binding protein 1; HuR, human antigen R; SOD2, superoxide dismutase 2; MyD88, myeloid differentiation primary response gene 88; AMPKα2, AMP-activated catalytic subunit alpha 2; RAC1, Rac family small GTPase 1; PAK2, p21-activated kinase 2; CDKN1B, Cyclin Dependent Kinase Inhibitor 1B; TGF-β, transforming growth factor-β; PDGF, platelet derived growth factor; DLL4, delta-like 4; Myo1e, myosin 1E; NLRP3, nod-like receptor protein 3; N/A, not available; BCL11B, B-cell chronic lymphocytic leukemia (CLL)/lymphoma 11B; Map4k4, mitogen-activated protein 4 kinase 4; JAM-A, junctional adhesion molecule A; Keap1, Keleh-like ECH-associated protein 1*.

#### ECs

ECs form the main components of the intima and act as a barrier between blood and tissues. A growing number of studies suggest that ECs-derived EVs are important in the pathogenesis of atherosclerosis (2). In addition, EVs are implicated in altering lipid metabolism and homeostasis, both of which are important for atherosclerosis.

MiRNAs in ECs-derived EVs play essential roles in atherosclerosis. MiRNA-126 packaged in EVs from ECs transferred to recipient cells can promote ECs proliferation, migration, and reendothelialization through modulating the target protein sprouty-related, EVH1 domain-containing protein 1 (SPRED1). A study analysis of 176 patients with coronary artery disease with and without diabetes mellitus found that the expression of miRNA-126 was decreased in EVs from diabetic patients (24). ECs-derived EVs can promoted anti-inflammation by reducing the expression of ICAM-1 through the transfer of miR-222 into recipient cells. EVs from high glucose-treated ECs exhibit a downregulated level of miR-222 and reduced anti-inflammation (30). It has been reported that EVs released from ECs can attenuate atherosclerosis by inhibiting inflammation. When transferred into monocytes, miRNA-10a in EVs from ECs exert anti-inflammatory effects by down-regulating the production of pro-inflammatory genes, including IRAK4, MAP3K7, and BTRC, and inhibiting the nuclear factor-kappa B (NF-κB) signaling pathway (22). MiR-126 and miR-210 of EVs were upregulated in ECs overexpressing hypoxia-inducible factor (HIF)-1. The transfer of miR-126 and miR-210 inhibited macrophages infiltration and reduced plaque area (3). EVs derived from human umbilical vein ECs (HUVECs) under stimulus, such as shear stress, exhibit a high level of miRNA-143/145 (27). Hergenreider et al. found that ECs-derived EVs overexpressed shear-responsive transcription factor Krüppel-like factor 2 (KLF2) induces miRNA-143/145 accumulation in ECs. Subsequently, EVs from ECs transfer miRNA-143/145 to VSMCs, thereby mediating the communication between ECs and VSMCs. Moreover, they found that overexpression of KLF2 in ECs attenuates the size of atherosclerotic plaque in ApoE(-/-) mice (27). In addition, miRNA-126 was enriched in EVs secreted by ECs. It promotes G protein-coupled receptor (GPCR) signaling by suppressing the function of the regulator of G protein signaling 16, thereby enhancing the production of CXC chemokine ligand 12 (CXCL12) (25). EVs produced from ECs containing miRNA-126 repress the amounts of macrophages and apoptotic cells in plaques, and increase the amount of VSMCs, resulting in less inflammation and small lesions. Ohta et al. reported that elevated IL-6 levels suppress the expression of miRNA-126-3p in EVs derived from ECs and promote monocyte cell line THP-1 cells adhesion, eventually enhancing intercellular adhesion molecule-1 (ICAM-1) levels, leading to exacerbation and deterioration of atherosclerotic plaques. In contrast, elevated the expression level of miRNA-126-3p has the opposite effects (26). The level of miRNA-19b was upregulated in EVs from hypoxia-induced ECs. The transfer of EVs MiRNA-19b can inhibit migration and angiogenesis of ECs through decreasing the expression of transforming growth factor-β2 (TGF-β2) (23). Lombardo et al. found that IL-3 increases the secretion of EVs and transmission of miRNA-126-3P from ECs to wounds by promoting the activity of signal transduction and activator of transcription 5 (STAT5), thereby stimulating angiogenesis (72). Additionally, ECs-derived EVs modulate ECs function, vascular angiogenesis through the delivery of miRNA-214 between ECs (73). van Balkom et al. reported that miRNA-214 enriched in EVs inhibits ataxia telangiectasia mutation and promote angiogenesis. Suppressing miRNA-214 levels shows opposite effects (73). On the contrary, miRNAs encapsulated in EVs from ECs can damage the function of blood vessels and accelerate atherosclerosis progression. Atherosclerosis associated inflammation enhances inflammatory factor accumulation (74), including tumor necrosis factor-α (TNF-α), which promotes the expression of miRNA-155 in ECs-derived EVs. MiRNA-155 of EVs downregulates endothelial Nitric oxide synthase (eNOS) expression and weakens endothelium vasorelaxation. Low production of eNOS contribute to endothelial dysfunction and impair vascular homeostasis (75). Furthermore, simvastatin has been shown to reduce the expression of miRNA-155 in EVs and improve eNOS generation (28, 76). In addition, miRNA-505 in EVs from ox-LDL treated HUVECs have been shown to enhance atherosclerotic development in mice models through impairing blood vessels function. Mechanistically, EVs released from ox-LDL-induced HUVECs co-cultured with neutrophils transfer miRNA-505 to neutrophils, activate the NF-κB pathway and inhibit SIRT3 in neutrophils, resulting in reactive oxygen species (ROS) accumulation and increased formation of neutrophil extracellular traps (NETs) (31).

#### VSMCs

Vascular media is mainly composed of VSMCs, which are important in maintaining physiological functions and structural integrity of blood vessels. VSMCs proliferation is vital in plaque repair and might be a beneficial process in early atherosclerosis, while VSMCs death and senescence induce plaque instability and promote atherosclerosis (77).

VSMCs-derived EVs exert significant effects in regulation of inflammation, cell migration, vascular calcification and cell coagulation among others. Additionally, EVs secreted by VSMCs can be elevated through stimulation by platelet derived growth factor (PDGF) and TNF-α, leading to VSMCs calcification. VSMCs-derived EVs that are rich in the miRNA-143/145 cluster play important roles in modulating VSMCs differentiation, quiescent vs. proliferative phenotype and vascular homeostasis (78). Climent et al. reported that transforming growth factor-β (TGF-β) or vessel stress-treated VSMCs-derived EVs mediate ECs angiogenesis and vascular stability through the transfer of miR-143/145 (79). Besides, molecules such as TNF-α and PDGF-BB enhance production of miRNA-143 in EVs, resulting in cell proliferation, cell migration, and VSMCs calcification (3, 80, 81). MiRNA-143-3p rather than miRNA-143-5p is enriched in EVs from pulmonary artery smooth muscle cells (PASMCs), and regulated PASMCs migration. Deng et al. revealed that miR-143-3p packaged in EVs derived from PASMC paracrine to ECs and act as a proangiogenic regulator (80). In addition, EVs from VSMCs transfer miRNA-150 to ECs, enhancing the expression of vascular endothelial growth factor-A (VEGF-A) and promoting the VEGF-A/VEGFR/PI3K/Akt pathway, leading to ECs migration (35). Besides, miRNA-150 knockdown in VSMCs attenuate ECs migration (35). Studies have shown that miRNA-150 overexpression induced by either spliced X-box binding protein 1 (XBP1) or PDGF-BB is crucial in regulating ECs and VSMCs function as well as in maintaining vessel homeostasis (35, 82). Dysregulation of autophagy by ECs has an effect on the progression of atherosclerosis. MiRNAs in EVs bind autophagy-related genes to inhibit ECs autophagy. Human aortic SMCs co-cultured with HUVECs were shown to suppress the autophagic activity of HUVECs through upregulating the levels of miRNA-221/222 in EVs from human aortic SMCs. Phosphatase and tensin homolog deleted on chromosome 10 (PTEN) is an anti-inflammatory and antifibrotic modulator, mediating VSMCs differentiation, migration and proliferation (83). The elevated expression levels of miRNA-221/222 in EVs downregulated the expression of PTEN in HUVECs and activated the Akt pathway, resulting in LC3II, Beclin-1, ATG5 downregulation and SQSTM1/p62 upregulation (37). Furthermore, atherosclerosis is associated with downregulated miRNA-133a levels (84). Gao et al. reported that miRNA-133a levels were decreased in ApoE(-/-) atherosclerosis mice models and were associated with inhibited insulin-like growth factor-1 receptor (IGF-1R) levels as well as suppressed VSMC growth (33). In addition, overexpression of miRNA-133a targets proapoptotic genes, including *Bim (Bcl2l11)* and *Bmf (Bcl-2 modifying factor)*, inhibiting cell apoptosis and promoting proliferation of cardiac progenitor cells (34). On the contrary, overexpression of KLF5 in VSMCs induces miRNA-155 production and secretion (36). KLF5 plays a role in promoting VSMCs proliferation by upregulating the generation of growth factors such as PDGF-A (85). VSMC-derived EVs transfer miRNA-155 to ECs, impairing EC barriers by weakening tight junctions and coherence, leading to increased endothelial permeability and enhanced atherosclerotic development (36). Moreover, miRNA-92a and miRNA-195 in EVs released from VSMCs shuttled into macrophages to regulating inflammation and promoting atherosclerosis through elevating the expression levels of IL-6 and CXCL1 (32).

#### Monocytes/Macrophages

Monocyte-endothelial cell adhesion induces numerous monocyte progression to macrophage- or DC- like phenotypes in early atheromata. Risk factors such as hyperlipidemia and hypertension can trigger vascular inflammation and further stimulate the expression of adhesion molecules such as ICAM-1, VCAM-1, and selectin E in ECs. These molecules promote monocyte-ECs adhesion and monocytes infiltration into the subcutaneous space of ECs (86). In early atheromata, the majority of monocyte progression to macrophage- or DC- like phenotypes (87). Many macrophages and dendritic-like cells have cytoplasmic membrane-bound lipid droplets (foam cells). Foam cells secrete numerous pro-inflammatory cytokines and promote monocyte proliferation and accumulation. Accumulating data suggests that macrophages regulate atherosclerosis development by destabilizing atherosclerotic plaques. Ox-LDL induces monocytes/macrophages inflammatory response emerge as a key event in atherosclerosis.

EVs released from macrophages stimulate pro-inflammatory responses by transporting miRNAs and lncRNAs into recipient cells (88). For example, M1-like-type macrophages secrete numerous pro-inflammatory EVs, with anti-angiogenic properties (45). Besides, M2-like-type macrophage-derived EVs have both pro-inflammatory and anti-inflammatory roles (89). Monocyte/macrophage-derived EVs can modulate cell proliferation and migration, inflammation, apoptosis and angiogenesis. Zhang et al. reported that miRNA-150 is transferred from THP-1 cells to ECs through EVs. Mechanistically, THP-1 cells-derived EVs were shown to have an abundance of miRNA-150, which are transferred to human microvascular ECs-1 (HMECs-1), resulting in HMECs-1 migration by downregulating c-Myb (43). Notably, c-Myb, a nuclear transcription factor, was found to be highly expressed in atherosclerotic mice models and acted as a critical regulator of the atherosclerosis process (90). In addition, miRNA-21-3p and miRNA-142a-5p of EVs are secreted by macrophages. Macrophages exposed to nicotine trigger the transfer of miRNA-21-3p to VSMCs *via* EVs, and induce VSMCs proliferation and migration by directly inhibiting PTEN (38). EVs containing miRNA-106a-3p can be transferred from ox-LDL-treated THP-1 macrophages to VSMCs, thereby promoting VSMCs proliferation and inhibiting cell apoptosis by combining with CASP9 to modulate the Caspase signaling pathway. Contrastingly, treatment with EVs secretion inhibitor, GW4869, suppresses EVs secretion from ox-LDL-induced THP-1 cells, decreasing VSMCs viability and increasing cell apoptosis (40). Wang et al. reported that the transfer of miRNA-222 in EVs from M1 macrophages to VSMCs elevated VSMCs proliferation and migration through the mediation of Cyclin Dependent Kinase Inhibitor 1B (CDKN1B) and CDKN1C *in vitro* and enhanced neointima formation after carotid artery injury *in vivo*, which was reversed by 2'OMe-miR-222, a miRNA-222 inhibitor (46). Human THP-1 macrophages exposed to ox-LDL were shown to exhibit elevated expression levels of miRNA-155 in a dose-dependent manner. MiRNA-155 from THP-1 cells is an atheroprotective regulator that inhibits inflammatory responses during the pathogenesis of atherosclerosis (44). Silencing miRNA-155 in THP-1 cells significantly promotes lipid intake, upregulates the secretion of scavenger receptors, leading to the accumulation of inflammatory factors such as IL-6, IL-8, and TNF-α. Furthermore, attenuation of miRNA-155 activation significantly elevates myeloid differentiation primary response gene 88 (MyD88) levels and suppresses the NF-κB pathway. M1 macrophage-derived EVs carrying miRNA-155 shuttle into ECs, playing an anti-angiogenesis role by inhibiting the activity of Sirtuin 1/protein kinase AMP-activated catalytic subunit alpha 2 (AMPKα2), endothelial nitric oxide synthase and Rac family small GTPase 1 (RAC1)/p21-activated kinase 2 (PAK2) signaling pathways (45). MiRNA-210 is a critical anoxic-related miRNA in EVs from cardiac progenitor cell. It promotes angiogenesis and suppresses myocardial cells apoptosis (91). Moreover, HIF-1α in inflammatory and lesional macrophages increases miRNA-210 expression and decreases miRNA-383 production, reduces ATP levels and elevates necroptosis and atherosclerosis through targeting 2,4-dienoyl-CoA reductase and mitochondrial ROS accumulation (92). Moreover, miR-210-3p suppresses the NF-κB pathway by inhibiting IGF2/IGF2R, resulting in amelioration of lipid congestion and inflammation (93). Furthermore, differentiation of macrophages induces EVs secretion and stimulates miRNA-233 delivery to recipient cells. Elevated miRNA-233 levels promote macrophages differentiation and apoptosis (94). It has been reported that miR-223, which is enriched in macrophage-derived EVs, acts as an inhibitor of Pknox1, thereby repressing macrophages inflammatory responses and downregulating adipose tissue inflammation (47). IL-4 induced naive bone marrow macrophages-derived EVs exhibit high expression levels of miRNA-99a/146b/378a, as well as downregulate NF-κB and TNF-α signaling pathways. Therefore, macrophages are polarized toward the M2-like-type, which ameliorates atherosclerosis lesion areas (39). On the contrary, miRNA-146a in EVs derived from macrophages can accelerate the development of atherosclerosis. EVs produced by ox-LDL exposed macrophages are rich in miRNAs, such as miRNA-146a, miR-185, miR-128, miR-503, and miR-365. It has been reported that EVs contained miRNAs transferred from donor macrophages to naive recipient macrophages are involved in the pathogenesis of atherosclerosis. MiRNAs encapsulated in EVs, especially miRNA-146a, from atherogenic macrophages, inhibit recipient macrophages migration and promote macrophages accumulation in vessel walls by suppressing the expression of insulin-like growth factor 2 mRNA-binding protein 1 (IGF2BP1) and human antigen R or ELAV-like RNA-binding protein 1 (HuR), thereby accelerating atherosclerosis progression (41). It has been accepted that IGF2BP1 is associated with cell proliferation and growth while HuR is involved in cell proliferation, differentiation, apoptosis and senescence (95, 96). Zhang et al. found that miRNA-146a was enriched in EVs produced from THP-1 macrophages treated with ox-LDL. MiRNA-146a promoted atherogenesis by targeting superoxide dismutase 2 (SOD2) and increasing the production of ROS and NETs (42).

#### DCs

DCs, a group of antigen-presenting cells, are implicated in the pathogenesis of atherosclerosis. During the development of atherosclerosis, DCs play roles in anti-inflammation, antigen presentation, lipid metabolism, and efferocytosis. Normal arteries have been shown to have low levels of DCs while atherosclerotic tissues have been shown to have elevated DC levels, implying that DCs play a critical role in atherosclerosis (97).

EVs secreted by DCs stimulated by other antigens seem to be atherogenic. Mature DCs-derived EVs are associated with ECs inflammation and atherosclerosis progression through the NF-κB signaling pathway. MiRNA-16 and miRNA-21 were found to be enriched in EVs from bone marrow-derived DCs treated with low-intensity pulsed ultrasonography (LIPUS). DCs-derived EVs co-cultured with HUVECs can inhibit TNFα-induced inflammation by ameliorating the expression of ICAM-1 and vascular cell adhesion molecule (VCAM)-1, subsequently inhibiting the NF-κB signaling pathway (48). In addition, it has been admitted that miRNA-155 promotes inflammation while miRNA-146a suppresses it. DCs-derived EVs exhibit elevated levels of miRNA-146a and miRNA-155. MiRNA-146a and miRNA-155 packaged in EVs from DCs, uptaken by recipient DCs, are involved in intercellular communication and modulation of inflammatory responses (50). Zhong et al. co-cultured mature DCs-derived EVs with HUVECs and found that EVs from mature DCs promoted the expression of adhesion molecules, such as VCAM-1 and E-Selectin by a quick activation of NF-κB signaling pathway. Further study indicated that mature DCs-derived EVs shuttled miRNA-146a into HUVECs, inhibiting interleukin 1 receptor associated kinase, which repressed the second stimulation of HUVECs *via* EVs and inhibiting ECs inflammation (49). The expression of miR-203-3p in EVs was downregulated in ox-LDL-induced bone marrow-derived macrophages (BMDMs). Lin et al. found that the transfer of miR-203-3p targeted cathepsin S in bone marrow-derived macrophages to attenuate atherosclerosis development in mice (51).

#### Platelets

Platelets, inflammatory cells with a well-established role in hemostasis and thrombosis, play central roles in the progression of atherosclerosis. Vascular exposure to stimuli such as hyperlipidemia and hyperglycemia initiates platelet activation as well as interactions with ECs and leukocytes, which has been implicated in atherosclerosis.

Platelet-derived EVs interact with monocytes, ECs and VSMCs, thereby promoting atherosclerosis progression. EVs secreted by thrombin-activated platelets inhibit ECs inflammation, cell proliferation and migration, angiogenesis and atherogenesis through miRNAs. MiRNA-25-3p has been shown to be enriched in EVs derived from thrombin induced platelets in atherosclerosis mice models. Peripheral blood platelet-derived EVs inhibited the expression of Adam 10 and further suppressed coronary vascular ECs inflammation and lipid deposition through reducing the levels of IL-1β, IL-6, TNF-α, α-smooth muscle actin, Collagen I a1, Collagen III a1, total cholesterol, and triglycerides through the delivery of miR-25-3p. In addition, miRNA-25-3p packaged in EVs from platelets alleviates ECs inflammation by suppressing the NF-κB signaling pathway (52). Li et al. found that thrombin-activated platelet-derived EVs from atherosclerotic animal models were rich in miRNA-21, miRNA-223, and miRNA-339. MiRNA-223 transferred to HUVECs to suppressed ICAM-1 production, which can be reversed by a miRNA-223 inhibitor. Thrombin-activated platelets-derived EVs carry miRNA-223 and inhibit ECs inflammation by modulating MAPK and NF-κB signaling pathways (56). Moreover, platelets-derived EVs contain miRNA-223, which regulates ECs apoptosis by decreasing IGF-1R levels (55). The expression levels of miRNA-21, miRNA-223, and miRNA-339 are associated with platelet activation and are enriched in platelet-derived EVs. They inhibited co-cultured VSMCs proliferation by suppressing PDGFRβ production, which is associated with VSMCs invasion and migration (57, 98). Additionally, in acute coronary syndrome patients, miRNA-126 has been shown to be significantly elevated in platelet-derived EVs. Isolated EVs, incubated with HUVECs, promote cell proliferation and migration by increasing the production of angiogenic factors, such as VEGF, basic Fibroblast Growth Factor and TGF-β1 (53). Thrombin-activated platelets-derived EVs contained a high level of miRNA-142-3p and shuttled miR-142-3p into ECs to promoted ECs proliferation through targeting Bcl-2-associated transcription factor-1(BCLAF-1) and its downstream genes (54). MiRNA Let-7a was enriched in platelet-derived EVs. The transfer of miRNA let-7a from platelet to HUVECs promoted angiogenesis through downregulating the generation of thrombospondin-1 (THBS-1), an anti-angiogenic factor (99). MiR-320b secreted by activated platelets was slightly elevated in the circulation of myocardial infarction patients. Activated platelet-derived EVs transfer miRNA-320b to human microvascular endothelial cell-1 cells to mediate inflammatory responses through downregulating the expression of ICAM-1 (58, 84).

#### MSCs

MSCs with the characteristics of self-renewal and multipotent differentiation as well as the capability of anti-inflammation may be promising therapeutic strategies for atherosclerosis. For example, bone marrow-derived MSCs (BMSCs) enhance the generation of anti-inflammatory cytokines, such as TGF-β1 and IL-10 and inhibit the expression of pro-inflammatory cytokines, such as TNF-α and IL-6 (100). An increasing number of publications indicate that EVs secreted by MSCs are served as atheroprotective regulators through regulating intercellular communications.

MSCs-derived EVs are implicated in regulating cell proliferation, migration, and apoptosis, and alleviating atherosclerotic plaque formation *via* miRNAs. Expression levels of miRNA-30b are highly elevated in MSCs-derived EVs. Co-cultures with HUVECs revealed that EVs transfer miRNAs from MSCs into HUVECs and enhance angiogenesis by suppressing delta-like 4 (DLL4) levels, a negative modulator of angiogenesis (59, 101). In addition, human adipose MSCs-derived EVs exhibit high expression of miRNA-125a that inhibit the production of DLL4, leading to ECs angiogenesis and the improvement of endothelial tip cell formation (61). MiRNA-31 was up-regulated in EVs from endothelial differentiation medium-treated adipose-derived stem cells. EVs containing miRNA-31 shuttled from SCs into HUVECs to regulate cell migration and tube formation through suppressing HIF-1 (60). Overexpression of miRNA-126 in MSCs-derived EVs were shown to promote cell proliferation and migration by enhancing the production of EGF, VEGF, bFGF, and PDGF in Hypoxia/Reoxygenation-injured ECs, as well as MSCs. Besides, miRNA-126-3p was found to be enriched in EVs from human umbilical cord MSCs. In rat models, miRNA-126-3p was shown to enhance HUVECs proliferation, migration, and tube formation through the SPRED-1/PIK3R2/AKT/ERK1/2 pathways (102). In contrast, Wang et al. reported that MSCs-derived miRNA-125b transfer to VSMCs through EVs suppresses VSMCs proliferation and migration as well as neointimal hyperplasia by ameliorating the expression of myosin 1E (Myo1e) (62). MSCs-derived EVs carrying miRNA-145 shuttled into HUVECs. The transfer of miRNA-145 inhibits cell migration and ameliorates atherogenesis plaque development *in vivo* by suppressing junctional adhesion molecule A (JAM-A), which plays an essential role in maintaining endothelial barrier functions (66, 103). Furthermore, MSCs-derived EVs containing miRNA-126 shuttled into ECs to attenuated ECs apoptosis by increasing the activity of PI3K/Akt/eNOS signaling pathway (65). Additionally, miRNA-342-5p in EVs from adipose-derived MSCs inhibits HUVECs apoptosis and protects ECs function against atherosclerosis by inhibiting PPP1R12B expression (69). Moreover, miRNA-125b-1-3p in EVs was highly expressed in human adipose-derived MSCs and reduced B-cell chronic lymphocytic leukemia (CLL)/lymphoma 11B (BCL11B) levels in lymphocyte, leading to lymphocyte apoptosis in atherosclerotic arterial tissues and in amelioration of atherosclerosis (63). EVs secreted by BMSCs from aorta tissues of ApoE(-/-) atherosclerosis mice models were shown to contain significantly elevated miRNA-125b-5p levels, which induced the suppression of mitogen-activated protein 4 kinase 4 (Map4k4), down-regulated MMP-9 expression, inflammation, blood lipid accumulation and plaque size, as well as increased α-SMA expression, thereby alleviating apoptosis in atherosclerosis mice (64). Furthermore, MSC-derived EVs can also attenuate atherosclerosis by modulating inflammation. ECs endocytosis of EVs with highly expressed miRNA-512-3p from MSCs was shown to suppress ox-LDL induced EC injury, Caspase-3 activation and cell apoptosis, decreased the secretion of inflammatory cytokines such as oxidative factor MDA, TNF-α, IL-1β and IL-6, increased GSH-PX and SOD levels and promoted ECs proliferation. Mechanistically, the transfer of miRNA-512-3p in EVs from MSCs inhibits Keleh-like ECH-associated protein 1 (Keap1) to alleviate ox-LDL-induced ECs dysfunction (70). In addition, EVs released from MSCs transfer miRNA-let7 into macrophages, where they promote M2 macrophage polarization, inhibit M1 macrophage polarization and alleviates atherosclerotic plaque in ApoE-/- mice models through the miR-let7/HMGA2/NF-κB signaling pathway (71). Moreover, MSCs-derived EVs suppress macrophage infiltration in the atherogenesis plaque through the miR-let7/IGF2BP1/PTEN pathway (71). Furthermore, EVs containing miRNA-301 transferred from BMSCs can protect against atherosclerosis through inhibiting myocardial autophagy (68). BMSCs-derived EVs carrying miR-223 to suppress the expression of nod-like receptor protein 3 (NLRP3) and inhibit macrophages pyroptosis, thereby inhibiting the rupture of atherosclerotic plaques (67).

### LncRNAs in EVs From Different Cells in Atherosclerosis

In recent years, numerous studies have demonstrated that lncRNAs act as endogenous sponges to mediate the function and expression of miRNAs (104). Accumulating evidence suggests that EVs containing lncRNAs are delivered to recipient cells to participate in macrophage polarization and proliferation and migration of VSMCs (Table 2).

**Table 2:** Function of lncRNAs in EVs in atherosclerosis.

**LncRNAs**	**Donor cells**	**Recipient cells**	**Expression**	**Target /pathway**	**Effects**	**References**
RNCR3	ECS	VSMCs	↑	miR-185-5p/ KLF2 axis	Inhibit the development of atherosclerosis through promoting VSMCs proliferation and migration	(105)
LINC01005	HUVECs	VSMCs	↑	miR-128-3p/ KLF4 axis	Inhibit the development of atherosclerosis through promoting VSMCs proliferation and migration	(106)
MALAT1	HUVECs	Monocytes	↑	CD206↑, IL-10↑, IL-12↓	Promote atherosclerosis progression by promoting M2 macrophage polarization	(107)
	HUVECs	DCs	↓	Nrf2	Promote atherosclerosis development through inducing DCs maturation	(108)
	HUVECs	Neutrophils	↑	NETs↑	Accelerate atherosclerosis development	(109)
ZEB1-AS1	HUVECs	HUVECs	↑	miR-590-5p/ETS1	Accelerate atherosclerosis development	(110)
GAS5	ECs	ECs	↑	miR-26a	Accelerate the progression of atherosclerosis by enhancing ECs apoptosis	(111)
	Monocytes			Caspases↑		(112)

*ECs, endothelial cells; HUVECs, human umbilical vein endothelial cells; VSMCs, vascular smooth muscle cells; DCs, dendritic cells; KLF2, Krüppel-like factor 2; NF-κB, nuclear factor-kappa B; TNF-α, tumor necrosis factor-α; IL-6, interleukin-6; ETS1, E26 oncogene homolog 1; TLR4, toll-like receptor 4*.

EVs contain a variety of lncRNAs were associated with inflammation. Studies have revealed that lncRNA ZEB1-AS1 was involved in ECs apoptosis and macrophage inflammation by regulating the ZEB1-AS1/miR-942/HMGB1 axis (113). Chen et al. found that ZEB1-AS1 was enriched in EVs from ox-LDL-treated HUVECs, where it induced inflammation, proliferation, and apoptosis by mediating the expression of E26 oncogene homolog 1 (ETS1). ETS1 promotes the activation of the TGF-β/Smad signing pathway, however, further research reveals that ZEB1-AS1 knockdown reverses this effect. Therefore, ameliorating the expression of ZEB1-AS1 may be a promising and potential therapeutic strategy for atherosclerosis (110). In addition, Wang et al. investigated the levels of EVs and EVs carried lncRNA HIF1A-AS1 in 35 atherosclerosis patients and 28 healthy adults. The study found that EVs and HIF1A-AS1 of EVs were highly expressed in atherosclerosis patients. Therefore, HIF1A-AS1 in EVs can serve as a potential diagnostic biomarker for atherosclerosis (114). Shan et al. established that lncRNA-RNCR3 is elevated in ECs and VSMCs of mouse and human atherosclerotic plaque. RNCR3 knockdown in ECs and VSMCs leads to the accumulation of inflammatory molecules, impairs the function of ECs and VSMCs, and worsens atherosclerosis (105). Besides, alleviating the level of RNCR3 in ECs by RNase A or proteinase K reduces the proliferation and migration ability of VSMCs. HUVECs-derived EVs containing RNCR3 has emerged as a competing endogenous RNA of miR-185-5p, leading to an increase in KLF2 (115). EVs released by ox-LDL-induced HUVECs carried a high level of LINC01005. ECs-derived EVs promoted VSMCs proliferation and migration through the transfer of LINC01005. Mechanistically, LINC01005 acted as a sponge of miR-128-3p and elevate the level of KLF4 (106). LncRNA-MALAT1 was upregulated in lipopolysaccharide (LPS)-activated macrophages. Mechanistically, MALAT1 interacts with the NF-κB signaling pathway to reduce the production of inflammatory cytokines (such as TNF-α and IL-6) through inhibiting its DNA binding activity (116). Huang et al. established that MALAT1 packaged in EVs was elevated in oxLDL-treated HUVECs. EVs carrying MALAT1 co-cultured with macrophages promoted M2 macrophage polarization, however, the loss of MALAT1 can reverse this effect (107). Rahman et al. reported that M2 macrophages play an essential role in inflammation and regression of atherosclerotic plaque (117). Moreover, EVs shed by oxLDL-induced ECs exhibited a high level of MALAT1. In ox-LDL treated HUVECs co-cultured with neutrophils, MALAT1 in EVs acts as a regulator increasing NETs formation and trigger hyperlipidemia and inflammatory responses, which in turn exacerbate atherosclerosis (109). Additionally, MALAT1 in EVs shuttles from ECs into recipient DCs and ameliorates the development of atherosclerosis. Overexpression of MALAT1 in EVs delivered to immature DCs inhibits ROS congestion and represses DCs maturation by interacting with the Nrf2 signaling pathway. Nrf2 is vital in modulating antioxidant gene expression and serves as an anti-inflammatory mediator (108, 118). Additionally, MALAT1 in EVs from HUVECs treated with ox-LDL exhibit lower expression compared with EVs from HUVECs without stimuli, an indication that the loss of MALAT1 in EVs promotes DCs maturation in atherosclerosis. Increasing evidence suggests that lncRNAs are significantly elevated in atherosclerotic plaque of patients and animal models and play an important role in atherosclerosis. LncRNA GAS5 is enriched in EVs from ox-LDL -induced THP-1 macrophages and it is more abundant in ECs, where they promote ECs apoptosis together with apoptotic factors such as caspases. GAS5 knockdown in THP-1 cells-derived EVs alleviates ECs apoptosis. In contrast, overexpression of GAS5 in THP-1 cells exposed to ox-LDL leads to THP-1 cell apoptosis, while GAS5 knockdown inhibits the apoptosis of THP-1 cells (112). Besides, Liang et al. pointed out that in patients with atherosclerosis and ECs treated with ox-LDL, the level of GAS5 was upregulated while miRNA-26a was downregulated. GAS5 can directly bind to miRNA-26a, resulting in ECs apoptosis and impaired autophagy flux (111). Notably, miRNA−26a-5p overexpression suppresses cell apoptosis by inactivating the TLR4/ NF-κB signaling pathway in human aortic ECs exposed to ox-LDL (119). Therefore, GAS5 in EVs derived from ox-LDL-induced THP-1 cells transferred into ECs causing cellular apoptosis *via* downregulation of miRNA-26a-5p and activation of the TLR4/ NF-κB pathways and the upregulation of apoptotic factors including Caspases.

## EVs Mediate Intercellular Communication Between Different Cells in Atherosclerosis

**Figure 2:**
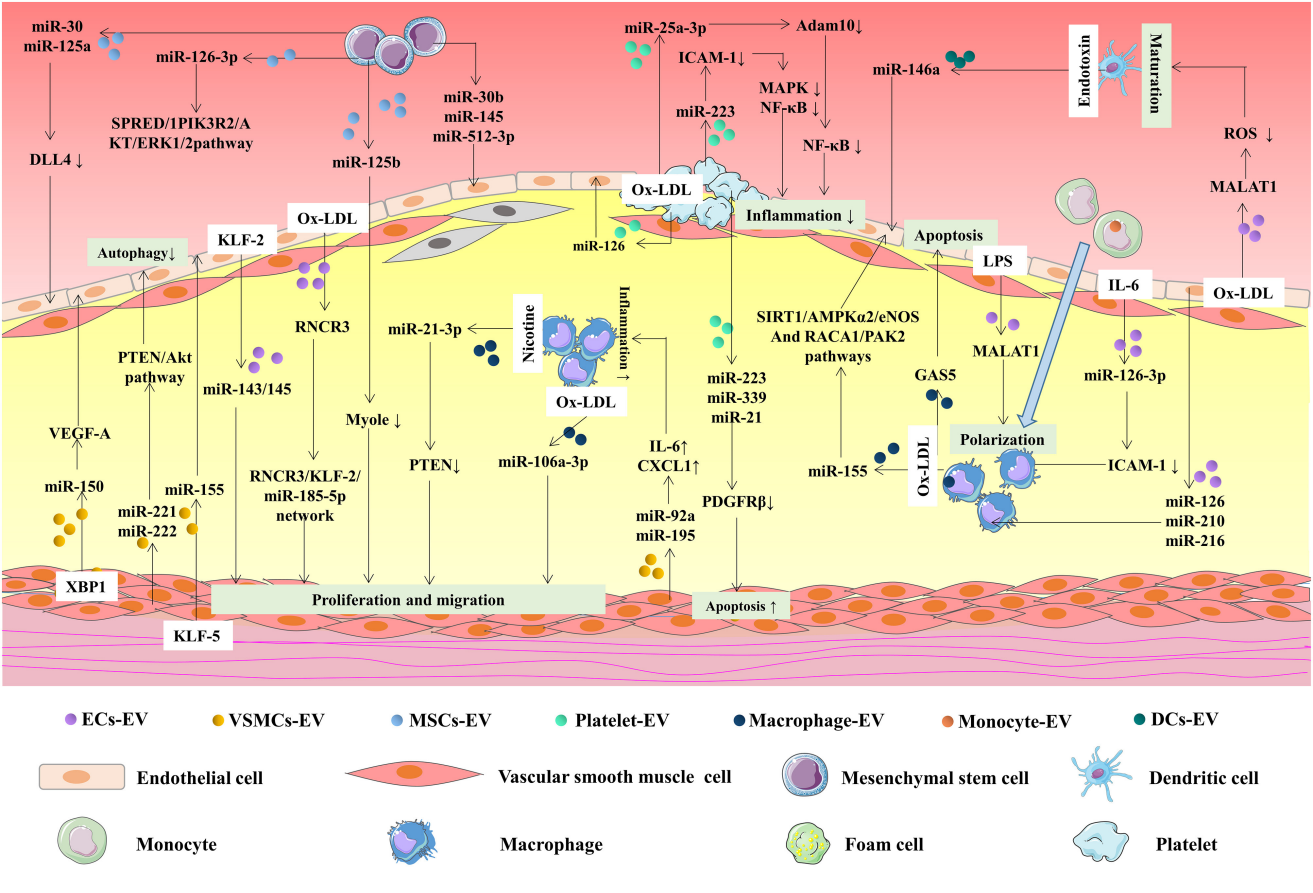
MiRNAs and lncRNAs in EVs mediate intercellular communication in atherosclerosis. EVs, secreted by different cells, including ECs, VSMCs, and immune cells, are implicated in atherosclerosis *via* transferring miRNAs and lncRNAs. KLF2-expressing ECs-derived EVs reduced atherosclerotic lesion formation in ApoE (-/-) mice *via* delivering miR-143/145. Ox-LDL-induced ECs secret EVs regulate VSMCs proliferation and migration through the delivery of RNCR3. On the contrary, XBP1 splicing in VSMCs can control ECs migration *via* EVs inclusion of miRNA-150 and miRNA-150-driven VEGF-A/VEGFR/PI3K/Akt pathway. MiR-221/222 in VSMCs-derived EVs repressed HUVECs autophagy *via* PTEN/Akt pathway. MiR-155 was elevated in KLF5-overexpressing VSMCs-derived EVs and enhanced atherosclerosis development *via* inhibiting ECs proliferation, migration, and re-endothelialization. ECs-derived EVs carrying miRNA-126, miRNA-210, miRNA-216, and MALAT1 alleviate macrophages polarization and infiltration. The transfer of miRNA-126-3P to macrophages can reduce atherosclerotic plaque area *via* EVs. Overexpression of miR-155 in EVs from ox-LDL-induced macrophages regulated ECs inflammation by modulating Sirt1/AMPKα2/eNOS and RAC1/PAK2 pathways. GAS5 was upregulated in THP-1 cells-derived EVs after oxLDL stimulation. GAS5 was involved in regulating ECs apoptosis and stimulating atherosclerosis. MiRNA-92a and miRNA-195 were increased in EVs from VSMCs and they could regulate inflammatory reaction through enhancing the expression of IL-6 and CXCL1 in macrophages. MiR-21-3p in EVs from nicotine-treated macrophages accelerate atherosclerosis by PTEN-mediated VSMC migration and proliferation. MiR-106a-3p was increased in EVs from ox-LDL stimulated THP-1 and it could promote cell proliferation of VSMCs. DCs-derived EVs transferred miRNA-146a into HUVECs to protect HUVECs from a second stimulation of TNF-α. Loss of MALAT1 in EVs from ox-LDL-induced VECs promoted DCs maturation in atherosclerosis development *via* Nrf2 pathway. MiR-25-3p was upregulated in thrombin-induced platelet-derived EVs and alleviated ox-LDL-induced ECs inflammation. Activated platelet-derived EVs promote ECs proliferation by transferring miRNA-126 and enhancing the production of VEGF, bFGF, and TGF-β1. Thrombin stimulated platelet-derived EVs repressed the production of PDGFRβ in VSMCs *via* the delivery of miR-223, miR-339, and miR-21, promoting cell apoptosis. MiRNA-30 and miRNA-125a in EVs released from MSCs reduced the expression of DLL4 in ECs, mediating angiogenesis. MSCs-derived EVs promote the proliferation and migration of ECs through delivering miRNA-126-3P. Besides, the transfer of miR-125b from MSCs-derived EVs to VSMCs inhibited VSMC proliferation by inhibiting Myo1e.

Atherosclerosis is a complex vascular disorder involving multiple pathological processes. ECs dysfunction, macrophages migration and foam cells formation, VSMCs proliferation and migration, and platelets activation contribute to the progression of atherosclerosis (120). A wide variety of stimuli trigger endothelial dysfunction to activate the NF-κB pathway, such as the release of numerous molecules, including ICAM-1, VCAM-1, and monocyte chemoattractant protein-1 (MCP-1) which are tightly associated with ECs dysfunction in atherosclerosis (121). Moreover, ICAM-1, VCAM-1, and NETs are implicated in leukocytes adhesion and migration to ECs and accumulating leukocytes and ECs secrete numerous inflammatory factors. Meanwhile, monocytes differentiate into macrophages and ultimately form foam cells (122). Besides, numerous chemokines and growth factors stimulate VSMCs proliferation and migration. EVs secreted by multiple cell types, including ECs, VSMCs, immune cells, and MSCs mediate intercellular communication in atherosclerosis. Accumulating evidence demonstrates that EVs, as well as EVs carrying miRNAs and lncRNAs, mediate cell-to-cell communication (Figure 2).

### MiRNAs and LncRNAs in EVs Mediated ECs-VSMCs Cross-Talk

ECs dysfunction in response to various stimuli such as hyperglycemia and hypercholesterolemia are central to atherogenesis. Notably, the NF-κB signaling plays an important role in ECs dysfunction, which is critical in the development of atherosclerosis. VSMCs proliferation and migration are important in atherosclerosis progression. ECs dysfunction is closely related to the function and migration of VSMCs through the delivery of EVs and their cargos. For example, our previous studies indicated that EVs from HUVECs exposed to high glucose induce VSMCs calcification (123). The intercellular communication between ECs and VSMCs through miRNAs and lncRNAs in EVs is bidirectional.

ECs-derived EVs mediate VSMCs phenotype. ECs treated with low or oscillatory shear stress in atherogenesis areas increase the activation of activator protein 1 (AP-1) and NF-κB signaling, leading to pro-inflammation and pro-coagulation during atherosclerosis. Several studies report that the level of KLF2 in ECs plays anti-inflammatory and anti-thrombosis roles in atherosclerosis by enhancing the generation of anti-inflammatory and anti-coagulant molecules, including thrombomodulin (TM) and eNOS. Moreover, KLF2 inhibits AP-1 and NF-κB activity (124). Emerging evidence supports that ECs in KLF2-/- show a normal endothelium, but dysfunctional and disorganized in VSMCs, indicating that KLF2 is involved in the migration of VSMCs (125). EVs secreted by HUVECs exposed to KLF2 or shear stress exhibit a high expression of miRNA-143/145 which is implicated in the control of VSMCs phenotype. The transfer of miRNA-143/145 from ECs to VSMCs modulate VSMCs migration and proliferation through EVs (27). Besides, lncRNAs in EVs have an impact on the progression and development of atherosclerosis. RNCR3 is highly expressed in ECs exposed to ox-LDL and human atherosclerotic lesions which are vital to atherogenesis. RNCR3 acts as an atheroprotective regulator *via* the RNCR3/ KLF2/miR-185-5p network. EVs containing RNCR3 from ECs transferred to VSMCs, mediates VSMCs proliferation and migration, and regulates VSMCs function giving rise to atherogenic phenotype (105). Besides, HUVECs stimulated VSMCs phenotype switch and proliferation *via* the transfer of LINC01005 in EVs (106).

VSMCs-derived EVs can influence ECs angiogenesis and vessel stabilization *via* miRNAs transfer. VSMC-derived EVs transfer miRNA-150 to ECs and trigger ECs migration by promoting the generation of VEGF-A and activation of the VEGF-A/VEGFR/PI3K/Akt pathway (35). Besides, co-cultured human aortic SMCs with ECs represses ECs autophagy activity by transferring miRNA-221/222 from human aortic SMCs-derived EVs (37). In contrast, EVs secreted by VSMCs with high expression of KLF5 deliver miRNA-155 to ECs, suppressing the proliferation, migration, and re-endothelialization of ECs. Besides, the transfer of miRNA-155 can enhance vascular endothelial permeability by destroying the tight junctions and integrity of endothelium barriers. KLF5, a zinc-finger-containing transcription factor, is vital to VSMCs proliferation and migration. Increasing evidence suggests that overexpression of KLF5 in VSMCs is significantly associated with ECs function during the process of atheromata (36).

### MiRNAs and LncRNAs in EVs Mediated ECs-Macrophages Cross-Talk

EVs mediate ECs and monocytes/macrophages communication *via* miRNAs and lncRNA. ECs exposed to various stimuli like ox-LDL trigger ECs injury and apoptosis, and recruit monocytes to adhere to ECs and differentiate into macrophages. Interestingly, ECs dysfunction induces inflammatory responses which are associated with monocytes/macrophages activation, eventually causing atherosclerotic plaque formation.

On the one hand, ECs-derived EVs can mediate monocyte activation and vascular inflammation. MiRNA-10a encapsulated in EVs transferred from ECs to monocytes, inhibits inflammatory responses (22). Additionally, miRNA-126-3P in EVs was decreased in IL-6-treated ECs, which increases the adhesion of THP-1 cells, while high levels of miRNA-126-3P show opposite effects (26). ECs-derived EVs inhibit macrophages accumulation and ameliorate atherosclerotic lesions through the delivery of miRNA-126 and miRNA-210 (29). Furthermore, MALAT1 was upregulated in EVs from ECs treated with ox-LDL and transferred into macrophages to induce M2 macrophage polarization (107). On the other hand, EVs derived from macrophages regulate ECs migration and angiogenesis. MiRNA-150 shed by EVs from THP-1 cells shuttled into ECs, thus promoting cell migration (43). Besides, EVs from M1 macrophages show an anti-angiogenesis effect on ECs. MiRNA-155 in EVs transferred from macrophages into ECs alleviated angiogenesis and stimulated cellular dysfunction (45). Moreover, the transfer of GAS5 in ECs-derived EVs modulates ECs and THP-1 cells apoptosis (112).

### MiRNAs and LncRNAs in EVs Mediated VSMCs-Macrophages Cross-Talk

EVs contained miRNAs and lncRNAs mediate the intercellular communication between VSMCs and macrophages during atherosclerotic progression. VSMCs-derived EVs carrying miRNA-92a and miRNA-195 transfer to macrophages and promote inflammation and atherogenesis (122). Moreover, EVs secreted by macrophages transfer miRNAs to VSMCs and mediate VSMCs function and migration. Nicotine-induced macrophages-derived EVs stimulated proliferation and migration of VSMCs and led to atherosclerotic development through the delivery of miRNA-21-3p (38). Besides, miRNA-106a-3p which is highly expressed in EVs from THP-1 macrophages treated with ox-LDL and incubated with VSMCs strengthens VSMCs proliferation and inhibits VSMCs apoptosis (40). Additionally, miRNA-222 significantly increases in M1 macrophages-derived EVs and are taken up by VSMCs, promoting VSMCs proliferation and migration through the target CDKN1B and CDKN1C (46).

### MiRNAs and LncRNAs in EVs Mediated DCs-ECs Cross-Talk

EVs mediate the communication between DCs and ECs, by inhibiting endothelial inflammation and preventing atherosclerotic deterioration. Existing evidence suggests that DCs-derived EVs are implicated in ECs inflammatory responses and atherogenesis through regulating NF κB signaling *via* TNF-α. MiRNA-16 and miRNA-21 of EVs isolated from DCs incubated with HUVECs suppress NF-κB pathway activation and repress endothelial inflammation associated with TNF-α (48). Moreover, the high level of miRNA-146a in mature DCs-derived EVs shuttled into HUVECs to protect HUVECs from a second stimulation by EVs from DCs, indicating that EVs from DCs play a negative role in mediating endothelial inflammation (49). Overexpression of MALAT1 in HUVECs-derived EVs secreted to DCs represses DCs maturation and attenuates ROS deposition. Preconditioning with ox-LDL, HUVECs exhibit low expression levels of MALAT1 promoting DCs maturation and atherosclerosis development (108).

### MiRNAs and LncRNAs in EVs Mediated Platelets-ECs and VSMCs Cross-Talk

EVs mediate the interaction among platelets, ECs, and VSMCs, inhibit endothelial inflammation and VSMCs proliferation, thereby, showing a protective effect in atherogenesis. Platelet-derived EVs containing miRNAs shuttle into ECs to modulate endothelial inflammation. It has been reported that miRNA-25-3p is significantly increased in EVs derived from thrombin-induced platelet and isolated EVs co-culture with coronary vascular ECs alleviate ox-LDL stimulated endothelial inflammation, lipid deposition, and fibrosis (52). EVs from platelets transfer miRNA-126 to HUVECs to improve migration and proliferation *via* upregulating angiogenic factors (53). Thrombin-activated platelets-derived EVs enhance ECs proliferation through the delivery of miR-142-3p (54). Platelets-derived EVs containing miRNA Let-7a can shuttled into HUVECs to increase angiogenesis (99). Platelets secreted EVs deliver microRNA-223 to ECs, and regulates cell apoptosis by attenuating IGF-1R expression (55). MiRNA-223 packaged in EVs shuttle into HUVECs alleviates endothelial inflammation by repressing the production of ICAM-1 and inhibiting MAPK and NF-κB pathways (56). Interestingly, platelet-derived EVs rich in miRNA-223, miRNA-339, and miRNA-21 uptake by VSMCs suppress cell proliferation by ameliorating the level of PDGFRβ (57).

### MiRNAs and LncRNAs in EVs Mediated MSCs-ECs and VSMCs Cross-Talk

EVs are involved in the exchange of information between MSCs and other cells, including ECs, VSMCs, and macrophages, regulating ECs angiogenesis and proliferation, VSMCs proliferation and migration, and macrophages polarization and infiltration. MiRNA-30b and miRNA-126-3P of EVs from MSCs delivered into recipient HUVECs increase cell tube-like structure formation and migration, and regulate angiogenesis (59, 102). Similarly, MSCs-derived EVs containing miRNA-125a, which enhances endothelial tip cell formation (61). Besides, SCs-derived EVs enhance ECs angiogenesis through transferring miRNA-31 (60). The delivery of miRNA-126 from MSCs to ECs promote ECs function *via* EVs (65). MiRNA-145 in EVs derived from MSCs alleviate HUVECs migration and reduce atherosclerotic plaque. Additionally, MSC-derived EVs containing miRNA-125b transferred to VSMCs ameliorates cell proliferation and migration by inhibiting Myo1e (62). MSCs-derived EVs shuttle into M2 macrophages, and induces cell polarization and infiltration through the transfer of miRNA-let7 (71).

## Application of miRNAs and lncRNAs in EVs in Atherosclerosis

Atherosclerosis is a major cause of high morbidity and mortality worldwide. Therefore, there is a need for studies to explore various diagnostic biomarkers and therapies for atherosclerosis. For early diagnosis, the identified biomarkers must be sensitive, precise, and specific differentiate atherosclerotic patients from healthy individuals (84). MiRNAs and lncRNAs packaged in EVs are indisputably implicated in the development, diagnosis, and potential therapy of atherosclerosis (120). Considering the increasing attention of EVs containing miRNAs and lncRNAs in atherosclerosis progression, they have emerged as novel diagnostic biomarkers and potential therapeutic targets for atherosclerosis.

### MiRNAs and LncRNAs in EVs as Diagnostic Biomarkers in Atherosclerosis

The roles of EVs carrying miRNAs and lncRNAs as potential biomarkers must, however, be carefully considered. For atherosclerosis diagnosis, the miRNAs include miRNA-192-5p, miRNA-16-5p, and miRNA-122-5p of EVs are reported to be reliable biomarkers for predicting intracranial atherosclerotic disease (126). Additionally, the high levels of miRNA-30e and miRNA-92a in EVs in 42 atherosclerosis patients, indicating that they may serve as promising diagnostic biomarkers for atherosclerosis (127). Evidently, miRNAs and lncRNAs in EVs are associated with atherosclerosis, including miRNA-21, miRNA-150, miRNA-155, miRNA-126, miRNA-210, miRNA-505, miRNA-146a, miR-133, miRNA-223, miRNA-125, miRNA-let7, RNCR3, MALAT1, GAS5, and HIF1A-AS1 which provide a strong basis for their application as diagnostic markers for atherosclerosis. Furthermore, the changes in expression of miRNAs and lncRNAs should be explored in the treatment of atherosclerosis, since they might provide promising prognostic biomarkers.

As for diagnosis, miRNAs and lncRNAs in EVs have important advantages of serving as non-invasive biomarkers due to EVs are easily accessible and highly stabile in body fluids, such as blood, saliva, and urine (88). In addition, miRNAs and lncRNAs encapsulated in EVs exhibit better application compared with circulating miRNAs and lncRNAs, due to the high sensitivity and specificity of EVs (128). MiRNAs of EVs are not affected by RNASE-dependent degradation. Therefore, they can stably detected in circulating plasma and serum, making them ideal biomarkers for clinical diagnostic applications (129). The level of miR-126 in EVs was positively correlated with the severity of coronary artery stenosis in acute myocardial infarction patients (130). Besides, miR-30e and miR-92a from plasma EVs could be used as novel biomarkers for atherosclerosis diagnosis (127). However, the application of miRNAs and lncRNAs carried by EVs as diagnosis biomarkers has not been clinically explored. Despite miRNAs and lncRNAs in EVs exert pivotal roles in the development, diagnosis, and potential therapy of atherosclerosis, there are still some limitations. First of all, the commonly used isolation technologies for EVs mainly included ultracentrifugation, size-based isolation techniques, and immunoaffinity capture techniques (131). Different EVs purification strategies may affect the content of miRNAs in EVs (132). Secondly, the large number of variable miRNAs carried by EVs may regulate various signaling pathways and have an overall effect on recipient cells. Therefore, it is difficult to thoroughly understand the function of miRNAs in EVs (133). Thirdly, it is difficult to identify miRNAs in a single EVs, or to measure the number of specific miRNAs carried by EVs when EVs are present in low abundance (134). Additionally, there is still no consensus on the optimal panel of EVs contained miRNAs or lncRNAs to use as diagnostic markers for atherosclerosis. The screening and development of the optimal panel require extensive and accurate studies involving substantial samples, which is time-consuming and labor-intensive. Therefore, there is a need to validate these miRNAs and lncRNAs packaged in EVs as clinical diagnostic and prognostic biomarkers in atherosclerosis using large cohort studies. More work is required in identifying the roles of miRNAs and lncRNAs in EVs as novel biomarkers in clinical management as well as therapies of atherosclerosis.

### MiRNAs and LncRNAs in EVs as Therapeutic Targets in Atherosclerosis

A better understanding of the underlying mechanisms of miRNAs and lncRNAs in EVs in atherosclerosis may allow for the development of EVs-based therapies against atherosclerosis. Numerous lines of evidence supports that EVs emerge as drug delivery carriers in the treatment of many disorders, for example, miRNAs-loaded EVs can pass through the blood-brain barrier and repress BACE1 in Alzheimer's disease (135). EVs can effectively transfer *Ldlr* mRNA to stabilize atherosclerosis plaques in *Ldlr*-/- mouse models (136). To date, there are over 200 preclinical studies associated with the role of EVs as therapeutic tools in different disease models (19). EVs and contained miRNAs and lncRNAs are tightly associated with atherosclerosis, thus, they might provide safe and reliable therapeutic targets for atherosclerosis treatment by overexpressing or knocking down prospective miRNAs and lncRNAs in EVs. Paeonol modulates monocytes-derived EVs inhibit NLRP3 activation and ameliorates the accumulation of inflammatory cytokines through the transfer of microRNA-223. Therefore, high expression of miRNA-223 in EVs might be a potentially novel strategy for the treatment of atherosclerosis (137). As discussed, miRNA-143/145 can also be explored in the development of potential treatment targets through mediating ECs and VSMCs communication and abrogating atherosclerotic plaque. Besides, upregulation of lncRNAs in EVs, including RNCR3 and MALAT1 provide a strong basis for their application as potential therapeutic targets. Reducing the expression of miRNA-126-3p, miRNA-146a, and miRNA-155 support fresh therapeutic strategies against atherosclerosis (26, 28).

With respect to clinical therapy, miRNAs and lncRNAs in EVs have the potential to become therapeutic targets or drug delivery systems for atherosclerosis. On the one hand, EVs have the ability to target tissues or cells and penetrate biological barriers (138). One of the biggest advantages of EVs is that they are endogenous and can avoid causing adverse events such as immune responses. Besides, EVs, as a drug delivery medium, have essentially biocompatibility and non-immunogenic compared to other nano-delivery systems. On the other hand, there exist several obstacles. One of the major problems in implementing EV-based therapies is the low productivity of EVs (139). Besides, natural EVs are unable to effectively deliver drugs or targeted applications due to their inadequacy, such as poor stability and rapid elimination (140). Moreover, natural EVs have the handicap of poor targeting. Therefore, the EVs used for treatment should be self-derived and modified to form engineered EVs, so as to avoid the side effects of exogenous EVs and compensate for the defects of natural EVs. Additionally, EVs cannot be stored for a long time which makes it difficult for transportation and clinical therapy. Thus, more studies are required to explore novel EVs preservation techniques (131). Nevertheless, it is important to consider the various unpredictable side effects. Therefore, miRNAs and lncRNAs in EVs delivery into intended sites is a challenging but interesting endeavor that requires further exploration.

## Perspectives and Conclusions

Atherosclerosis is implicated in intricate pathophysiological processes, including endothelial dysfunction, mononuclear macrophages infiltration, VSMCs proliferation and migration, and platelets activation. EVs secreted by multiple cell types have been demonstrated to participate in intercellular communication through the transfer of miRNAs and lncRNAs to recipient cells, inducing alterations in cell structure and function. Currently, numerous lines of studies have revealed the roles and mechanisms of miRNAs and lncRNAs in EVs as potential diagnostic biomarkers and therapeutic targets in atherosclerosis. In this review, we summarize the molecular and cellular mechanisms of different miRNAs and lncRNAs from diverse cells-derived EVs in the progression of atherosclerosis and discuss their potential applications in the diagnosis and treatment of atherosclerosis. Although the ability of EVs to deliver miRNAs and lncRNAs in atherosclerosis has aroused significant attention, there is a great need for more work to validate that miRNAs and lncRNAs in EVs serve as diagnostic biomarkers and therapeutic targets in atherosclerosis.

## Author Contributions

HX and Y-QN collected the literature and wrote the manuscript. Y-SL conceived the idea and supervised the manuscript. All authors read and approved the final manuscript.

## Funding

This work was supported by the National Natural Science Foundation of China (Nos. 82071593 and 81770833) and the Fundamental Research Funds for the Central Universities of Central South University (No. 2019zzts354).

## Conflict of Interest

The authors declare that the research was conducted in the absence of any commercial or financial relationships that could be construed as a potential conflict of interest.

## Publisher's Note

All claims expressed in this article are solely those of the authors and do not necessarily represent those of their affiliated organizations, or those of the publisher, the editors and the reviewers. Any product that may be evaluated in this article, or claim that may be made by its manufacturer, is not guaranteed or endorsed by the publisher.
